# The association between shift work and depression in hotel workers

**DOI:** 10.1186/s40557-015-0081-0

**Published:** 2015-12-12

**Authors:** Hyun Jey Moon, Sang Hyun Lee, Hee Sung Lee, Kyung-Jae Lee, Joo Ja Kim

**Affiliations:** Department of Occupational & Environmental Medicine, Soonchunhyang University Hospital, Seoul, South Korea

**Keywords:** Shift work, Shift type, Depression, Hotel workers

## Abstract

**Background:**

Shift work is vital in hotel businesses as these businesses run 24 h daily regardless of holidays to accommodate customers. The number of shift workers in hotel businesses is expected to increase consistently and it is crucial to study the impact of shift work on hotel workers’ mental health. This study, therefore, aims to examine the association between depression and shift work in hotel workers. It especially focuses on investigating whether there is a difference in how closely these two are related depending on shift types.

**Methods:**

A survey was conducted with 768 hotel workers who worked at two first-class hotels in Seoul. Out of 659 respondents total (response rate of 85.8 %), 506 respondents were selected as the final research subjects, excluding 153 respondents whose responses were incomplete. The survey was composed of questionnaires related to general characteristics, work-related characteristics, shift work, shift type, and depression level. The Korean Center for Epidemiologic Studies Depression (CES-D) Scale was used to evaluate the subjects’ depression level. Multiple logistic regression analysis was conducted with depression as a dependent variable and shift type as an independent variable after relevant general and work-related characteristics were adjusted to examine the relationship between shift type and depression.

**Results:**

After adjustment for relevant general and work-related characteristics, hotel workers had a significantly higher likelihood of belonging to the depression group than those with a fixed day shift, across all three shift types: rotating day shift (OR = 2.22, 95 % CI = 1.05–4.61), rotating night shift (OR = 2.63, 95 % CI = 1.11–6.24), and fixed night shift (OR = 3.46, 95 % CI = 1.02–11.74).

**Conclusions:**

The results showed that shift work was significantly related to depression in hotel workers and the risk of depression clearly differed among shift types. In particular, fixed night shift workers were most vulnerable to depression. Rotating day shift workers without night work could also have a higher risk of depression.

## Background

The “24-h society” has changed the timing of work towards longer and non-standard business hours. Because companies attempt to secure competitiveness by raising productivity to 24 h a day or extending business hours and as customer demands for out-of-hour services are increasing, flexibility in work hours has been requested. As a result, the arrangement of working hours has become a critical element in work organization, and the number of nontraditional working systems consisting of night work and shift work has been increasing [1, 2]. Although shift work was mainly found in the manufacturing industry in the past, the scope of shift work has extended to the retail and service industries due to globalization and development of information technology [3]. Approximately 15 ~ 18 % of employers are estimated to be involved in shift work in industrialized countries worldwide. Surveys show that 17.7 % of the United States’ workforce is estimated to have shift work [4]. In Europe, based on the result of Fourth European Working Conditions Survey published in 2007, shift workers made up about 17 % of the workforce [5]. The Ministry of Employment and Labor investigated the work-hour conditions of companies with 10 or more regular employees (3414 samples) in Korea in June 2011. The average rate of implementing the shift system for all industries was 15.2 % [6].

Shift work is vital in hotel businesses as these businesses run 24 h daily regardless of holidays to accommodate customers. Recently, more hotels are opening supplementary facilities such as in-room dining, bars, pools, and gyms for 24 h to accommodate higher customer demands for such services along with front desk service. Consequently, the ratio of shift work in hotel workers is high, and various types of shift work exist including continuous day shift without night work, rotating night shift, every-other-day (24-h) shift, and fixed night shift. According to the results of the previously mentioned 2011 survey on working hours in Korea, the shift system was introduced at a rate of 34 % in the accommodation and food service industry, more than twice the introduction rate in all industries (15.2 %) and similar to the rate found in a European Union survey [5, 6]. In addition, the growth of hotel businesses in Korea due to increased number of tourists from foreign countries is noteworthy. In 2012, the number of foreign inbound tourists to Korea was over 11 million. This number increased by 107 % compared to 5.32 million in 2000. This number increased by 6.2 % each year for the past 12 years [7]. In this case, it is only natural that the demands for hotels and hotel workers are greatly increasing. The country’s tourist accommodation business status estimates released by Tourism Knowledge and Information System of the Korea Culture and Tourism Institute showed that the number of tourist hotels increased from 621 in 2009 to 734 in 2013 and the number of the guest rooms increased from 67,171 to 79,393. Each number surprisingly increased by 18.2 % [8]. According to the Economically Active Population Survey of Statistics Korea (KOSTAT), the number of workers in the accommodation and food service industry, including hotels, increased by about 15 % in three years from 1.85 million in 2011 to 2.12 million in September, 2014 [9]. Therefore, high rates of shift work in the accommodation and food service industry are a global trend, and the number of hotel shift workers is expected to increase even more.

Shift work causes discordance between the endogenous circadian timing system and environmental synchronizers and interrupts the normal circadian rhythm [10, 11]. This gets workers to experience “jet lag,” which is a combination of feelings of fatigue, sleepiness, insomnia, disorientation, digestive troubles, irritability, decreased mental agility, and reduced performance efficiency. Besides these short-term effects, shift work also has long-term effects such as severe diseases that cause individuals and the society to spend significant amounts of financial and social cost [2]. Shift work is known to be related to physical disorders such as stroke [12], cardiovascular disease [13, 14], metabolic syndrome [15, 16], breast cancer [17, 18], and gastrointestinal disease [14, 19], and mental disorders such as sleep disorder [20, 21] and depression [22, 23]. Shift workers can also be isolated from their families and society; this is because most familial and social activities are based on the day-oriented rhythm of the general population, and therefore, shift workers find it difficult to participate in such activities [24, 25].

Depression is an important global public-health issue, because its lifetime prevalence ranging from 2 % to 15 % is relatively high, and it is associated with substantial disability [26]. Rated as the fourth leading cause of disease burden in 2000, depression accounted for 4.4 % of total disability adjusted life years (DALYs). Projections indicate that after heart disease, depression is expected to become the second leading cause of disease burden by the year 2020 [27]. Depression is problematic not only in the general population but also in the working population. The result of research analysis on the rate of mental disorders in workers demonstrated that depression had the highest prevalence rate after simple phobia [28]. In addition, depression in workers causes a significant amount of business and social costs as it leads to the decrease of productivity. Recently, several studies reported the cost-effectiveness of treatment and intervention for depression. Early discovery of depression and the importance of intervention were emphasized [29, 30].

Shift work and depression have been examined in workers of various job categories. Although there was no significant difference in depression between shift workers and non-shift workers in a study of university hospital nurses [31], depression level in shift workers was significantly higher in studies conducted with security guards [32], automobile manufacturing plant workers [33], steel manufacturers [34], and police officers [35], showing an overall association between shift work and depression. However, previous studies conducted comparative analysis merely on shift workers and non-shift workers, thereby overlooking the possibility of a difference in the association with depression depending on the type of shift work within shift workers. Furthermore, these studies did not discuss the continuous day shift without night work, which is currently rising as an issue. In addition, studies have rarely been conducted on hotel workers, who are one of the most representative shift worker groups. Studies about the impact of shift work on health conditions, especially mental health conditions, of hotel workers are rarely found. As mentioned previously, the number of shift workers in hotel businesses is expected to increase consistently and it is crucial to study the impact of shift work on hotel workers’ mental health. Studies about depression with its high disease burdens are considered to be especially important.

Therefore, this research was to understand depression in hotel workers, to examine the association between shift work and depression, and to confirm whether different shift types affected how strongly these two were related to each other.

## Methods

### Study subjects

The surveys along with medical examinations were conducted on 768 hotel workers who worked at two first-class hotels in Seoul in 2014. Of 659 total respondents (response rate of 85.8 %), 506 respondents were selected as the final research subjects, excluding 153 respondents whose responses were incomplete.

### Data collection and measurements

The survey was conducted from June 2014 to September 2014 using systematic survey questions; the data was collected based on respondent’s self-report. The survey was composed of questions about general characteristics, work-related characteristics, shift work, shift type, and the depression level of the respondents. All surveys were conducted after explaining to the respondents the study objective and the possibility of publishing the study results and obtaining signatures on consent forms from those who agreed to participate.

1. General characteristics
  Gender, age, marital status, education, current smoking, alcohol drinking, physical activity, and body mass index (BMI) were included as variables for general characteristics. Marital status was defined as married and unmarried (included divorce, separation, bereavement, etc.). Subjects were distinguished based on the information that they are or are not currently smoking, drinking alcohol, or doing physical activities. BMI was calculated using the subjects’ weight (kg) and height (m), and the subjects were categorized into three groups of underweight, normal, or obese based on 18.5 kg/m^2^ and 25 kg/m^2^, which are the Asian-Pacific standards defined by WHO [36].
2. Work-related characteristics
  The departments of a hotel can be subdivided into rooms (front desk, doors, bell desk, concierge), housekeeping, laundry, kitchen, food and beverage (wait staff), back office (finance, sales, and promotion), grounds (maintenance and repair, electricity, etc.), and facilities (swimming pool, fitness, store, etc.). Based on this, considering work-related characteristics and customer response service, these were categorized into the following five groups: rooms, food and beverage/facilities, kitchen, housekeeping (including laundry), and back office (including grounds). Different weekly working hours were grouped into 40 h or below, 41–48 h, and 49 h or above for the analysis. These groups were created considering the data distribution, based on the fact that weekly legal working hours in Korea is 40 h, and the fact that the International Labor Organization defines working hours over 48 h to be long work hours [37]. Work duration was categorized into below 10 years, 10–19 years, and 20 years or above including working years in similar positions. Employment status was categorized into permanent positions and temporary positions. The respondents were asked to record their annual net income excluding tax, and net income of below 20 million won, 20–29 million won, 30–39 million won, and 40 million won or above were used considering the survey data distribution.
3. Shift and night work
  Respondents were analyzed after being divided into four groups based on shift work and night work. Group 1 was a “fixed day shift” (workers who work fixed regular hours without shift and night work), group 2 was a “rotating day shift” (workers who work shifts without night work), group 3 was a “rotating night shift” (workers who work shifts with night work), and group 4 was a “fixed night shift” (workers who work fixed hours at night).
  The respondents were defined to be shift workers if their response to the question “Do you work with the shift system?” was “yes.” Out of these respondents, two-shift workers, three-shift workers, every-other-day (24-h) workers, and irregular shift workers were considered to have a “rotating shift”, and fixed night workers were considered to have a “fixed night shift.”
  Whether he or she belongs to night worker or not is decided referring to the standards of “special health examinations for night-time workers” starting in January 2014 based on Occupational Safety and Health Act. Respondents who responded “yes” to at least one of the following questions: “Do you work from 10 p.m. to 6 a.m. the next day four times or more on average in a month?” and “Do you work from 10 p.m. to 6 a.m. the next day for 60 h or more on average in a month?” were defined to be night workers. Respondents who responded “no” for both of these questions were defined to be day workers. However, respondents who responded “no” for both questions but were selected to be the subjects of “special health examinations for night-time workers” were defined to be night workers.
4. Depression
  The Korean Center for Epidemiologic Studies Depression (CES-D) Scale [38] was used to evaluate depression level. The responses for each questionnaire were scored between 0 and 3 with questions 5, 10, and 15 were scored inversely. Cho et al. suggested scores of 21 and above as the optimal cut off point for local community dynamics; this cutoff has been used to define the depression group [38, 39].

### Data analysis

First, chi-square testing was used to determine differences in the distributions of general and work-related characteristics for each shift type. Then the relationships between general and work-related characteristics and depression were analyzed.

Second, depression was used as a dependent variable and shift type was used as an independent variable to investigate the relationship between shift type and depression. Multiple logistic regression analysis was conducted after the general and work-related characteristics that demonstrated a significant relationship to depression in the chi-square testing were adjusted. The significance level of all statistics was *p* < 0.05 and SPSS 14.0 was used for the statistical analysis.

## Results

### General and work-related characteristics of the study subjects

Out of the total 506 research subjects, 311 (61.5 %) subjects were males and 195 subjects were females (38.5 %). The average age was 37.7 (39.7 for males, 34.5 for females), and the largest age group of the subjects was the 30s, second largest was the 40s, third largest was the 20s or younger, and the smallest was the 50s or older. In terms of marital status, the married group (52.6 %) was larger than the unmarried group (47.4 %). For education, most subjects received a bachelor’s degree or above (47.4 %) or an associate degree (41.1 %). Of the subjects, 26.9 % smoked, 70.0 % consumed alcohol, and 67.8 % worked out. For BMI, 65.8 % had normal weight, 25.1 % were obese, and 9.1 % had low weight.

The following was the distribution of general characteristics based on shift types. There was no significant difference between the four groups in terms of gender, but these groups showed significantly different results based on age (*p* < 0.05). All four groups consisted mostly of subjects in their 30s, but the subjects in their 50s were more likely to have fixed day shifts (24.1 %) while the subjects in their 20s were less likely to have this type of shift (14.7 %). The subjects in their 50s were less likely to have rotating day shifts, rotating night shifts, and fixed night shifts; the rates was 9.7 %, 14.3 %, and 15.4 % respectively. In addition, the subjects in their 20s and 30s were most likely to have rotating day shifts and rotating night shifts with rates of 66.4 % and 59.6 % respectively. Conversely, the subjects in their 30s and 40s were more likely to have fixed day shifts and fixed night shifts with the rates of 61.2 % and 69.2 % respectively. In terms of marital status, married subjects tended to have fixed day shifts and fixed night shifts with rates of 61.8 % and 53.8 % respectively. Unmarried subjects were more like to have rotating day shifts and rotating night shifts with rates of 50.9 % and 57.1 % respectively (*p* < 0.05). For education, 51.8 % of the subjects with fixed day shifts and 56.0 % of the subjects with rotating night shifts received a bachelor’s degree or above while 47.8 % of the subjects with rotating day shifts and 65.4 % of the subjects with fixed night shifts received an associate degree (*p* < 0.05). Current smoking, alcohol drinking, physical activity, and BMI did not demonstrate any significant differences based on shift types (Table 1).

**Table 1:** General characteristics of the study subjects by shift type

Characteristics	Day shift		Night shift		Total (*n* = 506)
	Fixed (*n* = 170)	Rotating (*n* = 226)	Rotating (*n* = 84)	Fixed (*n* = 26)	
	N (%)	N (%)	N (%)	N (%)	N (%)
Gender					
Male	102 (60.0)	139 (61.5)	55 (65.5)	15 (57.7)	311 (61.5)
Female	68 (40.0)	87 (38.5)	29 (34.5)	11 (42.3)	195 (38.5)
Age (years)*					
≦29	25 (14.7)	64 (28.3)	25 (29.8)	4 ( 15.4)	118 (23.3)
30–39	62 (36.5)	86 (38.1)	25 (29.8)	9 (34.6)	182 (36.0)
40–49	42 (24.7)	54 (23.9)	22 (26.2)	9 ( 34.6)	127 (25.1)
≧50	41 (24.1)	22 (9.7)	12 (14.3)	4 (15.4)	79 (15.6)
Mean ± SD	40.1 ± 10.0	35.9 ± 8.9	36.7 ± 9.8	40.1 ± 9.0	37.7 ± 9.6
Marital status*					
Married	105 (61.8)	111 (49.1)	36 (42.9)	14 (53.8)	266 (52.6)
Unmarried	65 (38.2)	115 (50.9)	48 (57.1)	12 (46.2)	240 (47.4)
Education*					
High school or below	28 (16.5)	21 (9.3)	8 (9.5)	1 (3.8)	58 (11.5)
College^a^	54 (31.8)	108 (47.8)	29 (34.5)	17 (65.4)	208 (41.1)
University or above	88 (51.8)	97 (42.9)	47 (56.0)	8 (30.8)	240 (47.4)
Current smoking					
No	130 (76.5)	158 (69.9)	64 (76.2)	18 (69.2)	370 (73.1)
Yes	40 (23.5)	68 (30.1)	20 (23.8)	8 (30.8)	136 (26.9)
Alcohol drinking					
No	52 (30.6)	60 (26.5)	31 (36.9)	9 ( 34.6)	152 (30.0)
Yes	118 (69.4)	166 (73.5)	53 (63.1)	17 (65.4)	354 (70.0)
Physical activity					
No	66 (38.8)	63 (27.9)	29 (34.5)	5 ( 19.2)	163 (32.2)
Yes	104 (61.2)	163 (72.1)	55 (65.5)	21 (80.8)	343 (67.8)
BMI					
< 18.5	11 (6.5)	21 (9.3)	9 (10.7)	5 (19.2)	46 (9.1)
18.5–24.9	112 (65.9)	159 (70.4)	48 (57.1)	14 (53.8)	333 (65.8)
≧25	47 (27.6)	46 (20.4)	27 (32.1)	7 (26.9)	127 (25.1)

*p < 0.05 by Chi-square test

^a^a 2 ~ 3 year course college

Out of 506 research subjects, 170 subjects (33.6 %) had fixed day shifts, 226 subjects (44.7 %) had rotating day shifts, 84 subjects (16.6 %) had rotating night shifts, and 26 subjects (5.1 %) had fixed night shifts. For departments, the largest group of subjects were in food and beverage/facilities (43.3 %), the second largest was kitchen (25.5 %), the third largest was rooms (13.4 %), the fourth largest was back office (11.5 %), and the smallest group was housekeeping (6.3 %). For work duration, the largest group had 10 years or less with the rate of 38.1 %, the second largest was 10–19 years (31.4 %), and the smallest was 20 years or more (30.4 %). For weekly working hours, the average was 43.5 (44.6 for fixed day shift, 43.2 for rotating day shift, 42.9 for rotating night shift, and 40.9 for fixed night shift), and the largest worked 40 h or less (55.5 %), the second largest worked 41–48 h (28.7 %), and the smallest worked 49 h or more (15.8 %). The largest group for annual income was 40 million won or above with the rate of 34.0 %, the second largest was the 30 million won range (28.7 %), the third largest was the 20 million won range (26.9 %), and the smallest was below 20 million won (10.5 %). For employment status, 90.1 % of the subjects had permanent positions while 9.9 % of the subjects had temporary positions.

The following was the distribution of the work-related characteristics based on shift types. In terms of departments, back office (28.2 %) had a relatively high rate of fixed day shifts while food and beverage/facilities (54.0 %) and kitchen (32.3 %) had high rates of rotating day shifts. Rooms (38.1 %) had a relatively high rate of rotating night shifts, but food and beverage/facilities (84.6 %) had fixed night shifts most frequently (*p* < 0.001). For weekly working hours, all four shift groups worked mostly 40 h or less; the rate was especially high in the fixed night shifts (84.6 %), while the rate was relatively low in the fixed day shifts (47.1 %). These shifts had a relatively high rate of long time workers who worked for 49 h or more (21.8 %; *p* < 0.05). In terms of annual income, the income of the largest group with fixed day shifts was in the range of 30 million won (34.1 %), while 33.6 % of the subjects with rotating day shifts, 32.1 % of the subjects with rotating night shifts, and 65.4 % of the subjects with fixed night shifts earned 40 million won or more; for these three shifts, the largest group was 40 million won or more. The subjects with fixed night shifts had a rate that was exceptionally high (*p* < 0.001). Work duration and employment status did not show any significant differences based on shift types (Table 2).

**Table 2:** Work related characteristics of the study subjects by shift type

Characteristics	Day shift		Night shift		Total (*n* = 506)
	Fixed (*n* = 170)	Rotating (*n* = 226)	Rotating (*n* = 84)	Fixed (*n* = 26)	
	N (%)	N (%)	N (%)	N (%)	N (%)
Department*					
Rooms	10 (5.9)	24 (10.6)	32 (38.1)	2 (7.7)	68 (13.4)
F&B^a^/Facilities	50 (29.4)	122 (54.0)	25 (29.8)	22 (84.6)	219 (43.3)
Kitchen	35 (20.6)	73 (32.3)	19 (22.6)	2 (7.7)	129 (25.5)
Housekeeping	27 (15.9)	3 (1.3)	2 (2.4)	0 (0.0)	32 (6.3)
Back office	48 (28.2)	4 (1.8)	6 (7.1)	0 (0.0)	58 (11.5)
Work duration (years)					
< 10	60 (35.3)	91 (40.3)	36 (42.9)	6 (23.1)	193 (38.1)
10–19	52 (30.6)	76 (33.6)	23 (27.4)	8 (30.8)	159 (31.4)
≧20	58 (34.1)	59 (26.1)	25 (29.8)	12 (46.2)	154 (30.4)
Weekly working hours*					
≦40	80 (47.1)	129 (57.1)	50 (59.5)	22 (84.6)	281 (55.5)
41–48	53 (31.2)	65 (28.8)	24 (28.6)	3 (11.5)	145 (28.7)
≧49	37 (21.8)	32 (14.2)	10 (11.9)	1 (3.8)	80 (15.8)
Mean ± SD	44.6 ± 5.8	43.2 ± 4.9	42.9 ± 4.4	40.9 ± 2.3	43.5 ± 5.1
Income (\10,000/year)*					
< 2000	9 (5.3)	28 (12.4)	16 (19.0)	0 (0.0)	53 (10.5)
2000–2999	51 (30.0)	67 (29.6)	16 (19.0)	2 (7.7)	136 (26.9)
3000–3999	58 (34.1)	55 (24.3)	25 (29.8)	7 ( 26.9)	145 (28.7)
≧ 4000	52 (30.6)	76 (33.6)	27 (32.1)	17 (65.4)	172 (34.0)
Employment status					
Permanent	154 (90.6)	204 (90.3)	72 (85.7)	26 (100.0)	456 (90.1)
Temporary	16 (9.4)	22 (9.7)	12 (14.3)	0 (0.0)	50 (9.9)

*p < 0.05 by Chi-square test

^a^Food and beverage

### Differences of general and work-related characteristics by depression

The Korean CES-D Scale was used to evaluate the depression level of the subjects. Subjects who scored 21 points or more were defined to be the depression group. Out of 506 research subjects, 83 subjects (16.4 %) belonged to the depression group. In general characteristics, gender, age, marital status, and physical activity showed statistically significant relationships with depression. The depression group number was significantly higher in females than males (*p* < 0.05), younger subjects (*p* < 0.05), unmarried subjects (*p* < 0.05), and subjects who did not participate in physical activities (*p* < 0.05). Education, current smoking, alcohol drinking, and BMI did not show any statistical significance (Table 3).

**Table 3:** Differences of general characteristics by depression

Characteristics	Normal group	Depression group^a^	*P* ^b^
	N (%)	N (%)	
Gender			
Male	271 (87.1)	40 (12.9)	0.007
Female	152 (77.9)	43 (22.1)	
Age (years)			
≦29	90 (76.3)	28 (23.7)	0.003
30–39	146 (80.2)	36 (19.8)	
40–49	114 (89.8)	13 (10.2)	
≧50	73 (92.4)	6 (7.6)	
Marital status			
Married	234 (88.0)	32 (12.0)	0.005
Unmarried	189 (78.8)	51 (21.3)	
Education			
High school or below	52 (89.7)	6 (10.3)	0.230
College^c^	168 (80.8)	40 (19.2)	
University or above	203 (84.6)	37 (15.4)	
Current smoking			
No	304 (82.2)	66 (17.8)	0.151
Yes	119 (87.5)	17 (12.5)	
Alcohol drinking			
No	134 (88.2)	18 (11.8)	0.069
Yes	289 (81.6)	65 (18.4)	
Physical activity			
No	127 (77.9)	36 (22.1)	0.017
Yes	296 (86.3)	47 (13.7)	
BMI			
< 18.5	39 (84.8)	7 (15.2)	0.141
18.5–24.9	271 (81.4)	62 (18.6)	
≧25	113 (89.0)	14 (11.0)	

^a^CES-D (center for epidemiologic studies-depression scale) score ≧21

^b^Calculated by Chi-square test

^c^a 2 ~ 3 year course college

In work-related characteristics, shift type, work duration, and income had statistically significant relationships with depression. The distribution of the depression group based on shift types showed that the rate gradually increased for fixed day shift (9.4 %), rotating day shift (19.0 %), rotating night shift (21.4 %), and fixed night shift (23.1 %) respectively (*p* < 0.05). For work duration, the depression group frequency was higher as the subjects had less work experience; 20 years or more was 8.4 %, 10–19 years was 18.9 %, and 10 years or less was 20.7 % (*p* < 0.05). In terms of income, the depression group number increased significantly as the subjects had lower income; 40 million won or above was 11.0 %, the 30 million won range was 17.2 %, the 20 million won range was 17.6 %, and below 20 million won was 28.3 % (*p* < 0.05). The order of largest to smallest distribution in the depression group in terms of departments was rooms (22.1 %), food and beverage/facilities (17.8 %), back office (15.5 %), kitchen (14.7 %), and housekeeping (3.1 %) respectively, but these did not have any statistical significance. The distribution of the depression group based on weekly working hours and employment status was not significantly different (Table 4).

**Table 4:** Differences of work related characteristics by depression

Characteristics	Normal group	Depression group^a^	*P* ^b^
	N (%)	N (%)	
Shift type			
Fixed day shift	154 (90.6)	16 (9.4)	0.022
Rotating day shift	183 (81.0)	43 (19.0)	
Rotating night shift	66 (78.6)	18 (21.4)	
Fixed night shift	20 (76.9)	6 (23.1)	
Department			
Rooms	53 (77.9)	15 (22.1)	0.177
F&B^c^/Facilities	180 (82.2)	39 (17.8)	
Kitchen	110 (85.3)	19 (14.7)	
Housekeeping	31 (96.9)	1 (3.1)	
Back office	49 (84.5)	9 (15.5)	
Work duration (years)			
< 10	153 (79.3)	40 (20.7)	0.005
10–19	129 (81.1)	30 (18.9)	
≧20	141 (91.6)	13 (8.4)	
Weekly working hours			
≦40	232 (82.6)	49 (17.4)	0.737
41–48	124 (85.5)	21 (14.5)	
≧49	67 (83.8)	13 (16.3)	
Income (\10,000/year)			
< 2000	38 (71.7)	15 (28.3)	0.026
2000–2999	112 (82.4)	24 (17.6)	
3000–3999	120 (82.8)	25 (17.2)	
≧ 4000	153 (89.0)	19 (11.0)	
Employment status			
Permanent	382 (83.8)	74 (16.2)	0.748
Temporary	41 (82.0)	9 (18.0)	

^a^CES-D (center for epidemiologic studies-depression scale) score ≧21

^b^Calculated by Chi-square test

^c^Food and beverage

### Association between shift type and depression

Simple logistic regression analysis was conducted with shift type as an independent variable and depression as a dependent variable. The result showed that the risk of being part of the depression group significantly increased for rotating day shift (OR = 2.26, 95 % CI = 1.23–4.17), rotating night shift (OR = 2.63, 95 % CI = 1.26–5.4 6), and fixed night shift (OR = 2.89, 95 % CI = 1.01–8.23) compared to the fixed day shift. Gender, age, marital status, physical activity, work duration, and income, which showed significant relationships to the distribution of the depression group in the cross analysis. Current smoking, alcohol drinking, and department were additionally adjusted to conduct a multiple logistic regression analysis. The variance inflation factors (VIF) of each independent variable were less than 10, presenting no problem in multicollinearity. After this was confirmed, the analysis was conducted. The result showed that the odds ratios gradually increased in the rotating day shift (OR = 2.22, 95 % CI = 1.05–4.61), rotating night shift (OR = 2.63, 95 % CI = 1.11–6.24), and fixed night shift (OR = 3.46, 95 % CI = 1.02–11.74) respectively compared to the fixed day shift and this result was statistically significant (Table 5).

**Table 5:** Odds ratios of depression by shift type

Independent variables	Unadjusted		Adjusted^a^	
	OR^b^	(95 % CI^c^)	OR^b^	(95 % CI^c^)
Shift type				
Fixed day shift	1.00	(reference)	1.00	(reference)
Rotating day shift	2.26	(1.23–4.17)	2.22	(1.05–4.61)
Rotating night shift	2.63	(1.26–5.46)	2.63	(1.11–6.24)
Fixed night shift	2.89	(1.01–8.23)	3.46	(1.02–11.74)

Calculated by multiple logistic regression analysis

^a^Adjusted for gender, age, marital status, current smoking, alcohol drinking, physical activity, department, work duration, and income

^b^ *OR* Odds Ratio

^c^ *CI* Confidence Interval

## Discussion

This research was to understand hotel workers’ depression, to confirm the relationship between shift work and depression, and to see whether the degree of relationship was different based on shift types.

First, the research subjects’ prevalence rate of depression was 16.4 %. This was lower than the rates of hotels and restaurants workers (28.8 %) [40], bankers (20.6 %) [41], and clinical nurses (36.3 %) [42] based on previous studies conducted in Korea using 21 points as the CES-D cut off point. The subjects of this research showed lower prevalence rates of depression because the subjects worked at the first class hotels and 90.1 % of these subjects were permanent employees. In addition, these subjects have relatively high incomes; 62.7 % of the subjects had an annual income of 30 million won or higher. Therefore, this result was due to the fact that job stability and appropriate compensation contribute to maintaining social and economic status and the fact that this eventually helps lower the depression level [43]. The prevalence rate of depression was higher in females (22.1 %) than in males (12.9 %) and the rate for females were significantly higher. When this result was compared with previous studies conducted in Korea using 21 points as the CES-D cut off point, the gender differences were greater than research conducted on Korean employees [40] with a rate of 14.7 % for males and 18.6 % for females and research conducted on bankers [41] with a rate of 19.8 % for males and 21.5 % for females. This research result is limited for direct comparison since it was not clinical major depression and was only point prevalence. However, population-based epidemiologic studies from 10 different countries, including Korea and the United States that compared lifetime rates of major depression [44] showed that females were two times more likely to have depression than males in all countries. This gender distribution was very similar to that of this study. In addition, depression level significantly increased with younger age, shorter work duration, and lower annual income. It is likely that younger age and shorter work duration make it difficult to adapt to work or result in establishing less mature relationships with employers or coworkers, thereby increasing fatigue levels and depression [45]. If one’s position is low due to short work duration, one’s control over the work is low while workload demands are high and income is relatively low. This can reduce one’s satisfaction with work and increase one’s depression level [43, 46]. From the perspective of hotel workers, their positions tend to be low when their age is younger and work duration is shorter. This increases their interaction with customers, which also increases their exposure to emotional labor and experience of violence, leading to increased risk of depression [47]. This is likely to be especially high in workers in charge of rooms and food & beverage whose main job is to interact with customers and satisfy their demands. Although there was no statistically significant difference in depression level according to department, the prevalence of depression was highest at 22.1 % and 17.8 % in rooms and food & beverage/facilities, respectively, while it was 3.1 % in housekeeping, which rarely involves interaction with customers. This shows that the risk of depression may differ according to work-related characteristics.

A comparison between the relationship of shift work and depression and the relationship based on shift types showed that when relevant general and work related characteristics were adjusted, the risk of being in the depression group was 2.22 times higher in the rotating day shift group, 2.63 times higher in the rotating night shift group, 3.46 times higher in the fixed night shift group compared to the fixed day shift. The result showed that the prevalence rate of depression is higher in the traditional rotating night shift. This corresponds to the results of studies previously conducted in Korea: night shift security guards were more likely to be depressed [32]; automobile manufacturing plant workers with shift work had a significantly higher Beck Depression Inventory score, which was a depression indicator [48]; and police officers with shift work were more likely to have depression [35]. This result also matches results of previous studies that showed that shift work including nighttime work negatively influences one’s depression level [22, 23].

The odds ratio of the rotating day shift was 2.22 and this had a significantly higher prevalence rate of depression compared to the fixed day shift. The odds ratio of the rotating night shift was 2.63 and this was not significantly different. This is meaningful because it confirms that there is a risk that shift work without night work may be related to depression. This is expected to be due to disruption of the circadian rhythm, which can cause mental health issues including depression, lack of familial and social support, and sleep disorders [2, 3]. Most subjects in the rotating day shift group had overlapping time with continuous two-shifts or three-shifts. Nine working hours, including one-hour mealtimes, were distributed between 06:00 and 22:00; the hours were different based on departments and hotels. Therefore, workers with morning work had to go to bed early to get to work in the morning with a sufficient amount of sleep. However, most personal activities took place at nighttime and it was difficult for these workers to reduce their participation in these activities. When they forced themselves to go to bed early, this put them close to circadian acrophase and made it difficult for them to initiate sleep [49]. Such working on a rotating day shift created significant sleep disturbances [21]. Compared to constant day workers, shift workers (with or without night shift) had a difficult time falling asleep [20]. Afternoon work or evening work less frequently interrupted circadian rhythm and caused sleep disturbance issues, but parents with evening work could not participate in child-related activities [50]. Similarly, evening workers could have reduced familial and social supports because they could not participate in social activities or family activities.

Lastly, the odds ratio of the fixed night shift was 3.46, which was the highest of all shift types; this result was caused by the decreased sleep duration and the maladjustment of the circadian rhythm. According to previous studies, fixed night workers seem to sleep somewhat less than rotating shift workers when averaged across the entire shift cycle [49, 51]. According to a review of the literature on the adjustment to fixed night work on the circadian rhythm in melatonin secretion, which is generally considered to be the best known indicator of the endogenous circadian body clock, a very small minority (<3 %) of fixed night workers show “complete” adjustment of their endogenous melatonin rhythm to night work, and less than one in four fixed night workers shown adjustment sufficiently “substantial” to derive any benefit from it [52]. There were no previous studies on the relationship between fixed night shifts and depression due to rarity of fixed night workers; there were only studies on the relationship between work injury [53] and work satisfaction level [54, 55]. Therefore, additional studies on the impact of fixed night work on mental health must be conducted to confirm this relationship. Any causal relationship must be revealed through conducting large-scale studies as the data for shift workers including fixed night workers are easily accessible due to the nighttime special health examination available since 2014.

In addition to shift work, numerous harmful occupational factors must be considered, because hotel workers serve in a variety of departments; in particular, workers in rooms and food and beverage/facilities who frequently interact with customers must be alert and attentive to their tastes and demands and are also required to maintain uncomfortable or stationary postures for long hours [47]. In the present study, the ratios of workers in rooms and food and beverage/facilities according to the type of shift work are fixed day shift (35.3 %), rotating day shift (64.6 %), rotating night shift (67.9 %), and fixed night shift (92.3 %), showing that shift workers are largely distributed in the department of rooms and food and beverage/facilities. Thus, for the association between shift work and depression in hotel workers, work-related characteristics such as emotional labor or experience of violence, which can occur with interaction with customers, must be considered. Efforts to better elucidate the association between shift work and depression in hotel workers by including such related risk factors in the model are required in future studies.

The limitations of this research are the following. First, this research is a cross-sectional study that does not provide any information about temporal relationship; it cannot investigate the causal relationship of shift work and depression. Second, this research was conducted on workers in two first-class hotels in Seoul and the depression level of the workers could have been underestimated because most of the research subjects were permanent employees. In addition, it is difficult to generalize to all hotel workers in Korea with this sample. Third, the measurement could be limited since each subject evaluated his or her own depression level when completing the survey. Finally, the “healthy worker effect” may interfere with the results of the study because the study was conducted on current employees and excluded retired workers who had health issues caused by shift work.

Despite such limitations, this research revealed that shift work and depression in hotel workers were related to each other and the differences were evident based on shift types. The depression risk was the highest with fixed night shift workers; this research showed that the depression risk of rotating day shift workers who did not have night work could also be increased. This research is noteworthy as it can be used to provide basic data for designing shift work types and developing preventative measures for mental disorders in hotel workers, especially since the number of the shift workers is expected to increase in this industry. More studies must be conducted on hotel workers’ shift work types, various factors related to shift work, for example, regularity, speed, and direction of shift rotation, and mental health including depression.

## Conclusions

This study showed that shift work and depression in hotel workers were related to each other and the differences were evident based on shift types. The depression risk was the highest with fixed night shift workers and the depression risk of rotating day shift workers who did not have night work could also be increased. These findings are noteworthy as it can be used to provide basic data for designing shift work types and developing preventative measures for mental disorders in hotel workers, especially since the number of the shift workers is expected to increase in this industry. This study also indicated the need for further research into various factors related to shift work, for example, regularity, speed, and direction of shift rotation, and mental health including depression.
